# Supplementary figures and images for: Low-cost intelligent surveillance system based on fast CNN
Source: PeerJ Comput Sci. 2021 Feb 25;7:e402. doi: 10.7717/peerj-cs.402 (PMC7959643; doi:10.7717/peerj-cs.402)

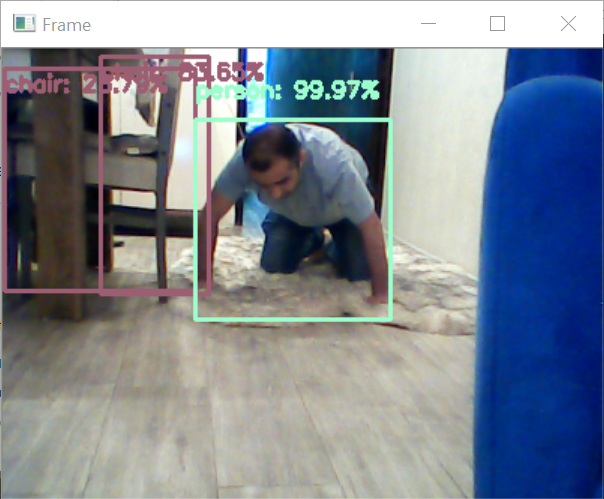

Supplement: Supplemental Information 1 [file peerj-cs-07-402-s001.zip › Recog. Results/8.PNG]

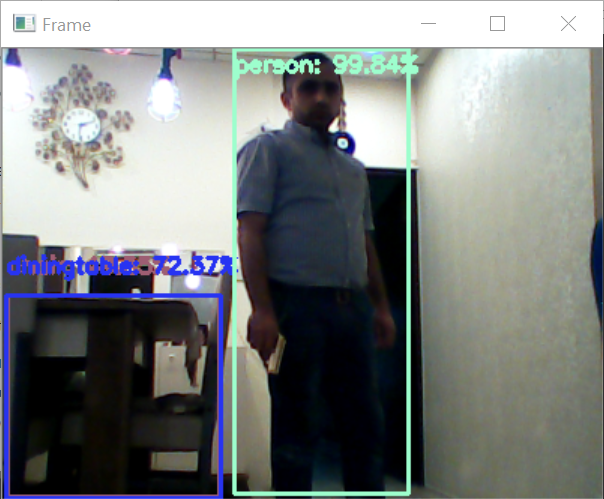

Supplement: Supplemental Information 1 [file peerj-cs-07-402-s001.zip › Recog. Results/9.PNG]

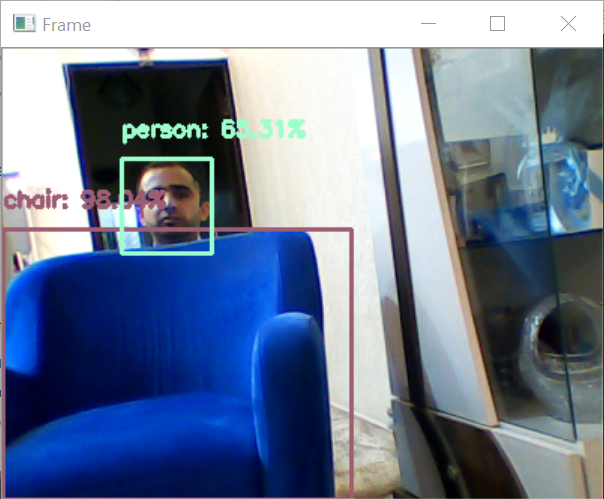

Supplement: Supplemental Information 1 [file peerj-cs-07-402-s001.zip › Recog. Results/4.PNG]

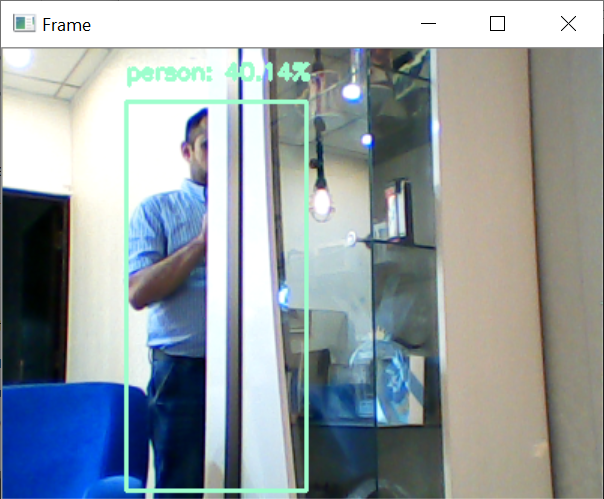

Supplement: Supplemental Information 1 [file peerj-cs-07-402-s001.zip › Recog. Results/5.PNG]

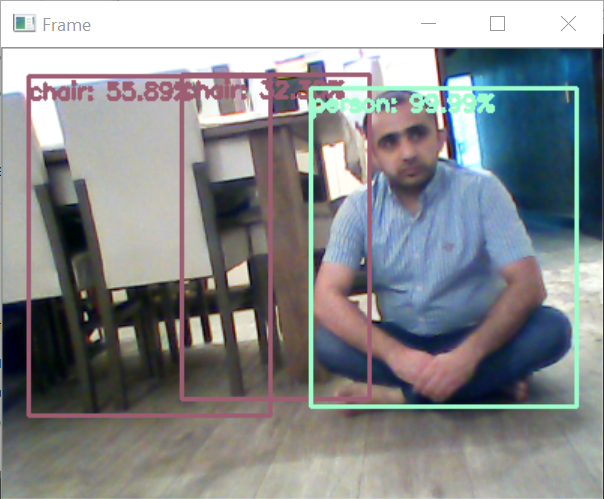

Supplement: Supplemental Information 1 [file peerj-cs-07-402-s001.zip › Recog. Results/7.PNG]

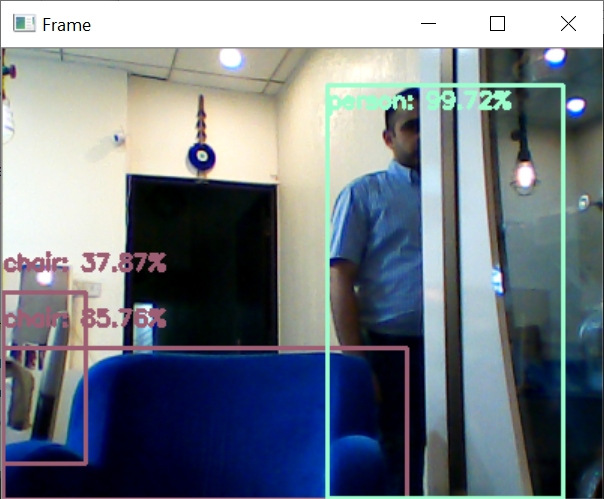

Supplement: Supplemental Information 1 [file peerj-cs-07-402-s001.zip › Recog. Results/6.PNG]

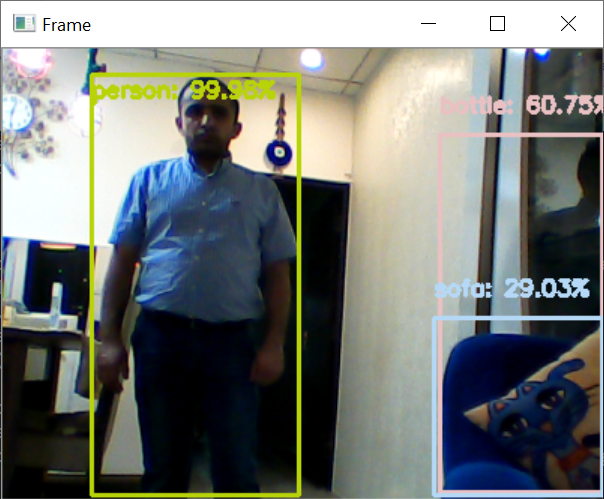

Supplement: Supplemental Information 1 [file peerj-cs-07-402-s001.zip › Recog. Results/2.PNG]

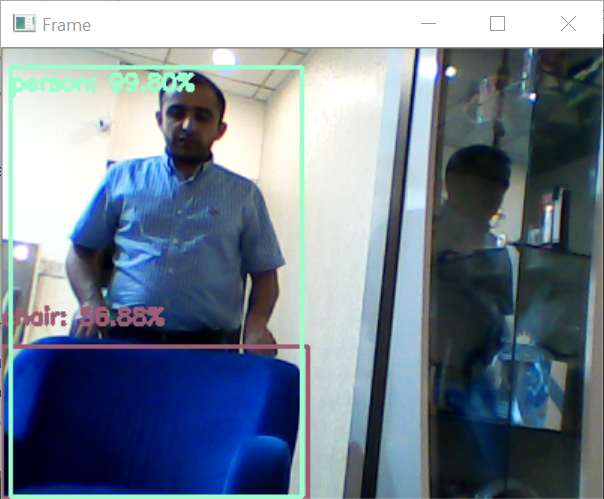

Supplement: Supplemental Information 1 [file peerj-cs-07-402-s001.zip › Recog. Results/3.PNG]

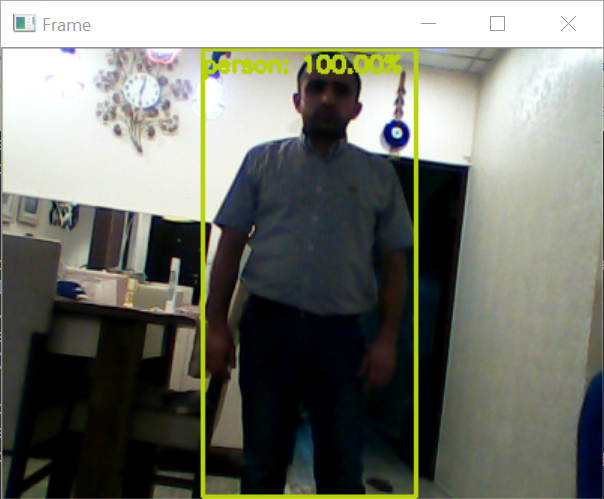

Supplement: Supplemental Information 1 [file peerj-cs-07-402-s001.zip › Recog. Results/1.PNG]
